# Changes in magnetic resonance imaging relaxation time on postmortem magnetic resonance imaging of formalin-fixed human normal heart tissue

**DOI:** 10.1186/s12880-021-00666-5

**Published:** 2021-09-23

**Authors:** Kiyokadzu Ebata, Sakon Noriki, Kunihiro Inai, Hirohiko Kimura

**Affiliations:** 1 Integrated and Advanced Medical Course, Graduate School of Medical Sciences, University of Fukui, 23-3 Matsuoka Shimoaizuki, Eiheiji-cho, Yoshida-gun, Fukui 910-1193 Japan; 2 Department of Radiology, University of Fukui Hospital, 23-3 Matsuoka Shimoaizuki, Eiheiji-cho, Yoshida-gun, Fukui 910-1193 Japan; 3 Faculty of Nursing and Social Welfare Sciences, Fukui Prefectural University, 4-1-1 Kenjojima, Matsuoka, Eiheiji-cho, Yoshida-gun, Fukui 910-1195 Japan; 4 Division of Molecular Pathology, Department of Pathological Sciences, School of Medical Sciences, University of Fukui, 23-3 Matsuoka Shimoaizuki, Eiheiji-cho, Yoshida-gun, Fukui 910-1193 Japan; 5 Department of Radiology, Faculty of Medical Science, University of Fukui, 23-3 Matsuoka Shimoaizuki, Eiheiji-cho, Yoshida-gun, Fukui 910-1193 Japan; 6 Autopsy Imaging Division, Education and Research Center for Medical Imaging, School of Medical Sciences, University of Fukui, 23-3 Matsuoka Shimoaizuki, Eiheiji-cho, Yoshida-gun, Fukui 910-1193 Japan

**Keywords:** Formalin fixation, Heart, Autopsy, Magnetic resonance imaging, Cause of death, Relaxation time

## Abstract

**Background:**

Postmortem magnetic resonance imaging (MRI) has been used to investigate the cause of death, but due to time constraints, it is not widely applied to the heart. Therefore, MRI analysis of the heart after formalin fixation was previously performed. However, the changes in MRI signal values based on the fixation time of formalin were not investigated. The objective was to investigate changes over time in the T1- and T2-values of MRI signals in normal areas of hearts removed during autopsy, hearts subsequently fixed in formalin, and heart specimens sliced for the preparation of pathological specimens.

**Methods:**

The study subjects were 21 autopsy cases in our hospital between May 26, 2019 and February 16, 2020 whose hearts were removed and scanned by MRI. The male:female ratio was 14:7, and their ages at death ranged from 9 to 92 years (mean age 65.0 ± 19.7 years). Postmortem (PM)-MRI was conducted with a 0.3-Tesla (0.3-T) scanner containing a permanent magnet. A 4-channel QD head coil was used as the receiver coil. Scans were performed immediately after removal, post-formalin fixation, and after slicing; 7 cases were scanned at all three time points.

**Results:**

The T1- and T2-values were calculated from the MRI signals of each sample organ at each scanning stage. Specimens were sliced from removed organs after formalin fixation, and the changes in T1- and T2-values over time were graphed to obtain an approximate curve. The median T1-values at each measurement time point tended to decrease from immediately after removal. The T2-values showed the same tendency to decrease, but this tendency was more pronounced for the T1-values.

**Conclusion:**

MRI signal changes in images of heart specimens were investigated. Formalin fixation shortened both T1- and T2-values over time, and approximation formulae were derived to show these decreases over time. The shortening of T1- and T2-values can be understood as commensurate with the reduction in the water content (water molecules) of the formalin-fixed heart.

**Supplementary Information:**

The online version contains supplementary material available at 10.1186/s12880-021-00666-5.

## Background

Postmortem imaging (PMI) using computed tomography (CT) and/or magnetic resonance imaging (MRI) to investigate the cause of death and elucidate the pathophysiology of a deceased person’s condition from internal images of the corpse came into use in the late 1990s [1, 2]. It is also known in different countries as “forensic radiology” [3] or “virtual autopsy” [4, 5] and, in Japan, it has been widely used and reported as “autopsy imaging” (Ai) since the turn of the millennium [6]. We ourselves have installed CT and MRI scanners specifically for postmortem use in a building adjacent to our university hospital, where we have engaged mainly in the Ai of autopsy patients from both within and outside the hospital, and we have conducted postmortem radiology-pathology correlation studies [7–11].

The commonly used PMI modalities include CT (PM-CT), as well as MRI (PM-MRI), ultrasound (US), and regular X-rays. Of these, CT is comparatively widely used. However, MRI generally provides better contrast resolution than CT, and since PM-MRI is capable of detecting conditions such as ischemic myocardium, muscle contusion, pediatric deformity, cervical spinal cord injury, and pulmonary artery thromboembolism that cannot be evaluated by superficial observations, given that PM-MRI can produce high-resolution images without the artifacts caused by body movements and vascular pulsation that are problems in clinical imaging, it is likely to improve the accuracy with which the cause of death can be established compared with PM-CT [12–19]. In the United Kingdom, PM-MRI has been introduced as an alternative to autopsy for investigating causes of death [20].

Myocardial infarction, one important cause of death, is difficult to diagnose because autopsy does not show any morphological changes in the heart within 6 h of onset. Cases of the detection of acute myocardial infarction in situ by 3-Tesla (3-T)-MRI [16] have been reported, as has the value of PM-MRI as a guide to autopsy in cases of sudden cardiac death [12]. Experiments on pig hearts have also shown that edema is detectable on T2-weighted imaging (T2WI) 3 h after myocardial infarction from total coronary artery occlusion [21].

Temporal limitations pose difficulties for PM-MRI of the human heart in situ. Because it is difficult to take about four hours for PM-MRI scanning before the autopsy, PM-MRI should be performed on formalin-fixed hearts after removal during autopsy.

Many studies involving MRI scanning of formalin-fixed organs have been published, but most studies addressed the relaxation time of brain tissue. Another study investigated changes in T1- and T2-values due to temperature changes in rat organs following formalin fixation after postmortem removal [22–36]. There has also been a study of the Achilles tendon [37].

In some studies, the T1- and T2-values of the formalin-fixed human heart were measured with MRI [38, 39]; the T1- and T2-values were measured, but the elapsed time after formalin fixation was not evaluated. The elapsed time after formalin fixation affects the MRI signal and is a very important matter.

The objective of the present study was to investigate changes over time in the T1- and T2-values of MRI signals in normal areas of hearts removed during autopsy, hearts subsequently fixed in formalin, and heart specimens sliced for the preparation of pathological specimens, as a basic study for the evaluation of heart disease by PM-MRI.

## Methods

### Subjects

The subjects were 23 consecutive pathological autopsy performed at our hospital from May 26, 2019 to November 16, 2020. Two cases were excluded because one case with a congenital heart malformation and one case of a 1-day-old newborn with an extremely small heart structure in which a signal measurement region of interest (ROI) of the same size as the other cases could not be set were excluded.

The male:female ratio was 14:7, and their ages at death ranged from 9 to 92 years (mean age 65.0 ± 19.7 years) (Table 1). Data on the causes of death and cardiovascular risk of the subjects were examined based on their medical records (Additional file 1: Table S1).

**Table 1:** List of autopsy and PM-MRI cases

Case no	Sex	Age (years)	Raw scan	Pre-cut scan (day)	Post-cut scan (day)
Case 01	M	59			○(216)
Case 02	M	72	○	○(10)	○(72)
Case 03	M	71	○		○(177)
Case 04	M	92			○(137)
Case 05	M	62			○(43)
Case 06	F	79		○(15)	○(81)
Case 07	M	78		○(23)	○(78)
Case 08	M	70	○	○(15)	○(67)
Case 09	F	76	○	○(18)	○(41)
Case 10	M	59	○	○(16)	○(53)
Case 11	F	59	○	○(14)	○(32)
Case 12	M	76	○	○(18)	
Case 13	M	20		○(14)	
Case 14	F	48		○(25)	
Case 15	F	9		○(46)	
Case 16	M	91		○(12,19.27,33,40)	○(59,82,108,121)
Case 17	F	75			○(6,15,25,34,49,71)
Case 18	M	75		○(3,5,7,10,12,14,26)	○(39)
Case 19	F	52	○	○(5,7,14)	○(37)
Case 20	M	72	○	○(3,5,8,10,17)	○(19,39)
Case 21	M	71	○	○(7,10)	

Raw scan: MRI scanned after the heart had been washed out with physiological saline immediately after removal

Pre-cut scan: MRI scanned with the formalin-fixed heart thoroughly immersed in formalin solution

Post-cut scan: MRI scanned after the fixed heart had been sliced, with the specimen closest to the papillary muscles chosen for scanning by the same method as the raw and pre-cut scans

M: F = 14:7 Age (y), mean (*SD*) = 65.0 ± 19.7

Pre-cut scan (day): scan day from death, mean (*SD*) = 15.4 ± 10.0

Post-cut scan (day): scan day from death, mean (*SD*) = 68.0 ± 49.6

This study was approved by The research ethics committee of University of Fukui (No. 20100064), and written, informed consent was obtained from the family of each deceased patient prior to autopsy for publication of the research and any accompanying images. A copy of the written consent is available for review by the Editor of this journal. This research conformed to the provisions of the Declaration of Helsinki. The datasets generated and/or analyzed during the current study are not publicly available.

### MR imaging

**Fig. 1:**
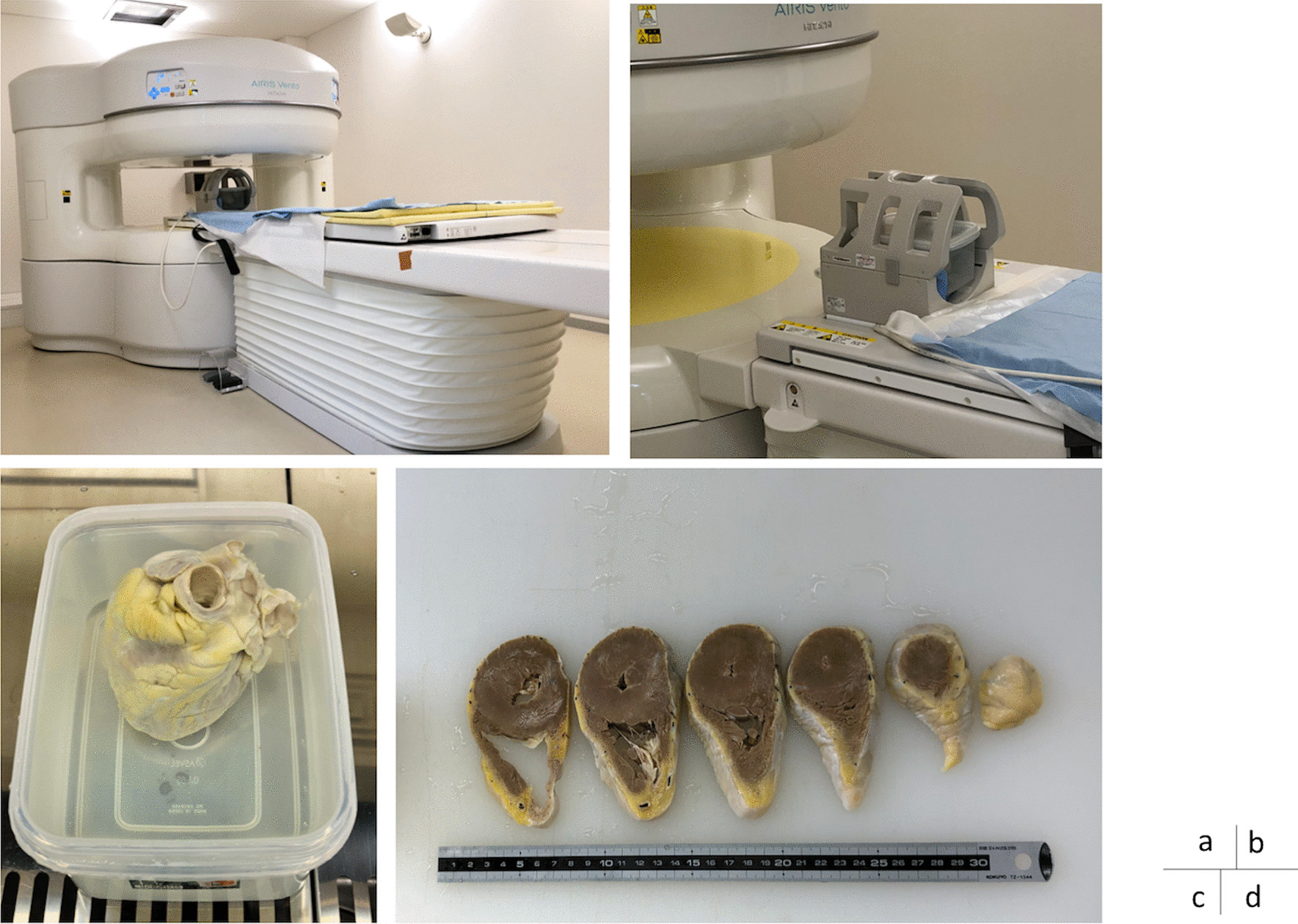
**a** External view of the MRI scanner. **b** QD coil. **c** Formalin-fixed heart. **d** Sliced heart

Postmortem MRI was conducted at the Ai center of the University of Fukui with a 0.3-T AIRIS Vento containing a permanent magnet (Hitachi Medical Corporation, Tokyo, Japan). A 4-channel QD head coil was used as the receiver coil (Fig. 1).

### Calculations

T1-values were calculated by the inversion recovery (IR) method. Inversion time (TI) was varied at 15 different time points between 20 and 2000 ms (ms), and the T1-values were calculated from the Bloch equation by the non-linear least squares method using equation [40] (1).

1 $$ {\text{M}}_{{\text{z}}} \left( \tau \right) = {\text{M}}_{0} \times \{ 1 - 2\;\exp \left( { - \tau /{\text{T}}1} \right)\} $$

The T2-values were calculated by the spin-echo method. The echo time (TE) was varied at 12 different time points between 10 and 200 ms, and the values were calculated using Eq. (2).

2 $$ {\text{M}}_{{\text{x}}} \left( \tau \right) = {\text{M}}_{0} \times \exp \left( { - \tau /{\text{T}}2} \right) $$

τ: TE, M_z_(τ): longitudinal magnetization over τ, M_0_: longitudinal magnetization at thermal equilibrium, M_x_(τ): transverse magnetization over τ.

The T1-value takes 6 min and 15 s per measurement, with an additional 15 s of pre-scanning required between each measurement. This measurement was performed 15 times. The T2-value takes 6 min and 28 s per measurement; for the T2-value, this measurement was performed 12 times. Before starting a series of MRI scans, images need to be taken to determine the position to scan and the cross-section of each specimen. This imaging series to determine the scan position took about 10–15 min each for one specimen, and since the measurement of T1- and T2-values was planned and measured as a series of consecutive scans, the start-to-finish imaging time per specimen was typically 3.5–4 h.

### Scan conditions

In order to make sure that the heart was sufficiently still in the container, a resting time was provided. In addition, time was set aside for the heart to remain in place for at least 30 min after being set in the scan position to ensure that the heart in the special container was shaken as little as possible in the solution by the movement of setting the container with the heart in the MRI gantry. After the resting time was secured, a series of measurements was started by changing TI and TE (Tables 2, 3). This sequence of procedures from setting the specimen to imaging was performed for all measurements investigated in this study.

**Table 2:** PM-MRI Parameters. MRI pulse sequence scanning conditions

	T1-value	T2-value
TR	3000	3000
TE	10.0	Table3 #2
TI	Table3 #1	–
FA	90	90
Thickness	5.0	5.0
FOV	200	200
Scan time	6′15″ × 15	6′28″ × 12

TR: time of repeat (millisecond)

TE: echo time (millisecond)

TI: inversion time (millisecond)

FA: flip angle (degree)

FOV: field of view (millimeter)

**Table 3:**
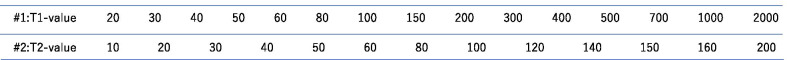
PM-MRI parameters. TI and TE assigned for signal acquisition to obtain T1 and T2-values

As mentioned at the beginning, the purpose was to measure the relaxation time of non-pathological parts of the heart structure. Therefore, it was necessary to confirm that the ROI was normal myocardial tissue.

In 11 cases, the blood was washed out of the heart with physiological saline immediately after its removal during the autopsy, after which the removed heart was immersed in physiological saline and scanned by MRI. This MRI scan immediately after removal was termed the “raw scan.”

In 16 cases, the heart was scanned by MRI under the same scanning conditions as the raw scan after it had been fixed by immersion in formalin, and this was termed the “pre-cut scan”. If time permitted, consecutive scans were performed under the same conditions. A total of 33 pre-cut scans of these 16 specimens were conducted.

In 16 cases, the formalin-fixed, removed heart was sliced into sections parallel to the annulus for the preparation of pathological tissue specimens, and the approximately 2-cm-thick slices were thoroughly soaked in formalin. They were then left for at least 6 h in the MRI gantry before being scanned under the same conditions as those used for the raw and pre-cut scans. These were termed the “post-cut scans.” If time permitted, consecutive scans were performed under the same conditions. A total of 25 post-cut scans of these 16 specimens were conducted.

In 7 patients, MRI was successfully performed at all three stages (immediately after removal, post-fixation, and after slicing).

### Region of interest designation

**Fig. 2:**
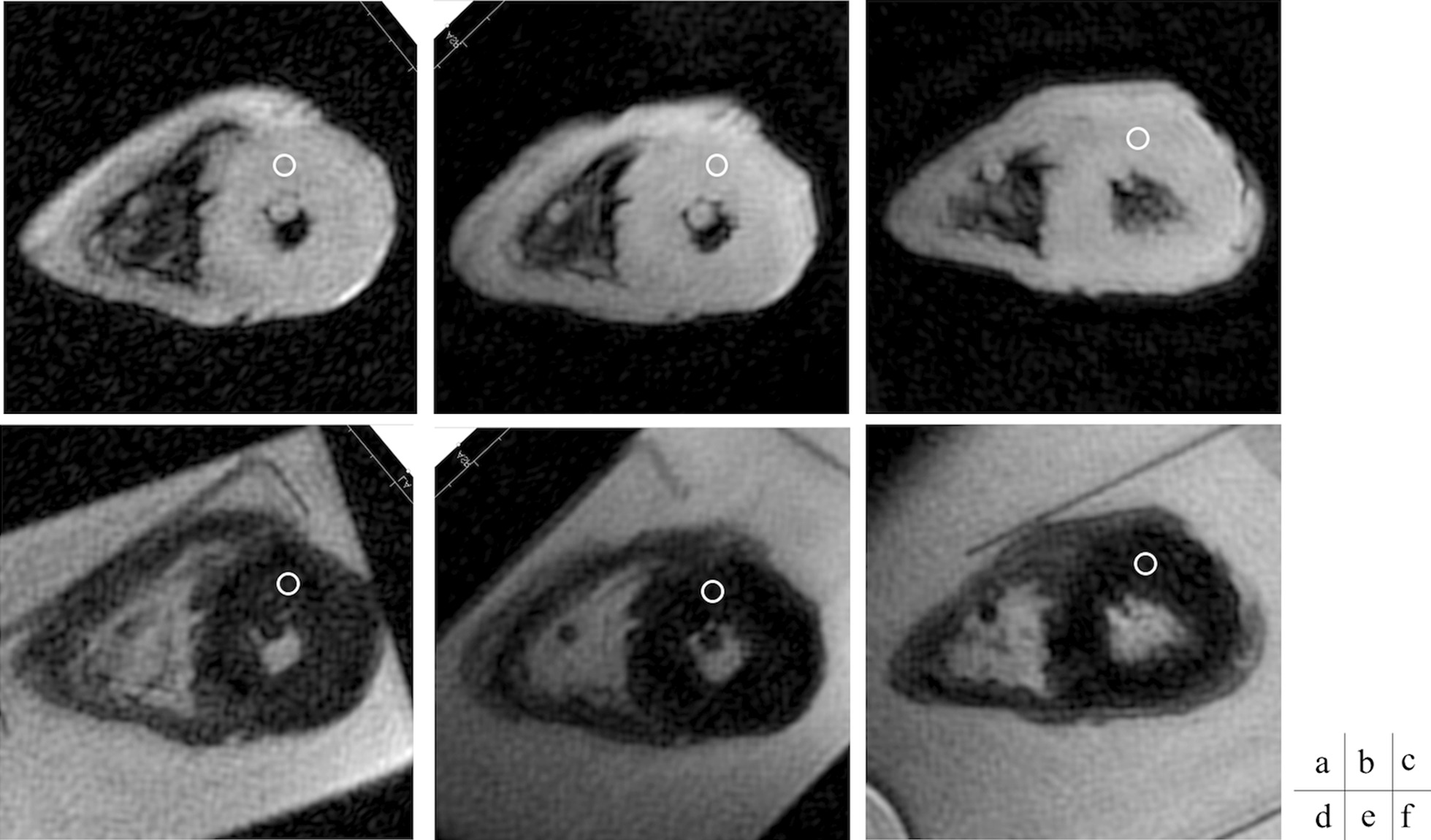
Case 9 **a** T1-value image Raw scan TI = 1000 ms. **b** T1-value image Pre-cut scan (18 days) TI = 1000 ms. **c** T1-value image Post-cut scan (41 days) TE = 200 ms **d** T2-value image Raw scan TE = 200 ms. **e** T2-value image Pre-cut scan (18 days) TE = 200 ms. **f** T2-value image Post-cut scan (41 days) TE = 200 ms

**Fig. 3:**
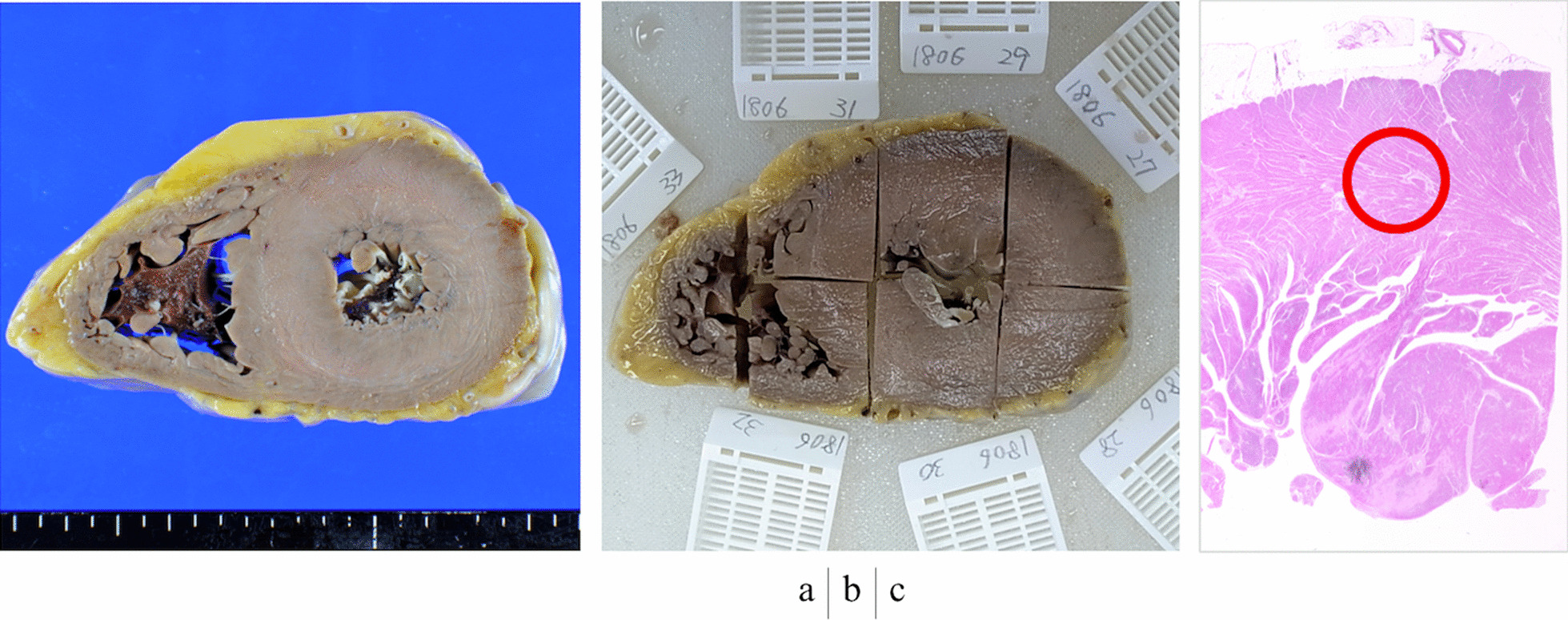
Case 9 **a** Sliced specimen. **b** Sampling. **c** Magnified view. The red circle indicates the ROI

The specimens were sampled, and regions of interest (ROIs) for signal measurement were designated in consecutive slices that had been microscopically confirmed by the pathologist to be structural tissue with no apparent pathological changes (Figs. 2, 3).

The ROIs used for all measurements were circular, 10 mm in diameter, in all cases. In addition, before measurement, the location of the ROI was always checked in detail for each specimen to ensure that it was the same location.

### Temperature management and temperature measurements

To investigate the effect of temperature changes before and after MRI scanning of the specimens, the temperatures of the MRI chamber and of the specimens before and after MRI scanning were measured. The MRI chamber was air-conditioned to a constant 23 °C for 24 h a day. Solution temperatures were measured using a Quick Check SN-820 core thermometer (Netsuken, Tokyo, Japan). The surface temperature of the specimens was measured using an infrared thermometer Model SK-8940 (Sato Keiryoki Mfg. Co., Ltd., Tokyo, Japan). A solution generator was used for the formalin solution, and a stable solution concentration was obtained.

### Statistical analysis

The accumulated data were stored in a database, operating behind a firewall, and password-protected. Data extracts were exported to Microsoft Excel (Microsoft Corp., Redmond, WA, USA). To check the normality of the distribution of the measured values of T1 and T2, the Kolmogorov–Smirnov test was performed and showed normal distributions. Then, the coefficient of determination (R^2^) was calculated. The sample size was calculated to be 20 cases at a significance level of 5%, power of 80%, and correlation coefficient of 0.6. All statistical analyses were performed with EZR (Saitama Medical Center, Jichi Medical University, Saitama, Japan), which is a graphical user interface for R (The R Foundation for Statistical Computing, Vienna, Austria) [41]. The relationships of the T1- and T2-values to the time course were fitted to the curve-fitting routine of Excel.

## Results

The T1- and T2-values were calculated from the MRI signals of each sample organ at each of the scanning stages (raw scan, pre-cut scan, and post-cut scan). The values obtained over time from organ removal were displayed graphically.

Raw scans: T1- and T2-values were measured in 11 samples of regions pathologically diagnosed as normal in hearts scanned by MRI immediately after removal. The shortest T1-value was 349.7 ms, and the longest was 453.3 ms; the median value was 411.7 ms, and the mean value was 407.5 ± 21.6 ms. The shortest T2-value was 62.4 ms, and the longest was 87.6 ms; the median value was 77.1 ms, and the mean was 77.6 ± 6.0 ms.

Pre-cut scans: The mean time from the start of fixation to scanning of the specimens scanned at this stage was 15.4 ± 10.0 days. The shortest time was 3 days, and the longest was 46 days. T1- and T2-values were measured in 16 specimens. The shortest T1-value was 193.8 ms, and the longest was 258.1 ms; the median was 226.7 ms, and the mean was 223.2 ± 16.0 ms. The shortest T2-value was 50.5 ms, and the longest was 62.5 ms; the median was 54.4 ms, and the mean was 55.2 ± 3.2 ms.

Post-cut scans: The mean time from the start of formalin fixation to scanning of the specimens scanned at this stage was 68.0 ± 49.6 days. The shortest time was 6 days, and the longest was 216 days. The shortest T1-value was 133.8 ms, and the longest was 221.8 ms; the median was 172.5 ms, and the mean was 175.4 ± 21.3 ms. The shortest T2-value was 47.3 ms, and the longest was 65.0 ms; the median was 51.6 ms, and the mean was 53.4 ± 4.7 ms (Table 4).

**Table 4:** T1- and T2-values of each case

Scan-timing	Case	T1-value	Median / mean ± SD	T2-value	Median/ mean ± SD
Raw scan	2	427.9	411.7/407.5 ± 21.6	77.1	77.1/77.6 ± 6.0
	3	411.7		78.7	
	8	453.3		87.6	
	9	374.3		69.0	
	10	383.5		76.0	
	11	418.5		62.4	
	12	409.7		73.9	
	17	349.7		74.5	
	19	403.4		84.6	
	20	421.7		82.7	
	21	428.4		87.2	
Pre-cut scan	2	213.3	226.7/223.2 ± 16.0	59.7	54.4/55.2 ± 3.2
	6	258.1		54.1	
	7	224.0		59.6	
	8	225.6		53.6	
	9	195.2		52.6	
	10	231.2		52.3	
	11	204.4		62.5	
	12	227.8		61.5	
	13	234.0		51.0	
	14	193.8		51.4	
	15	196.1		52.4	
	16*	208.2		54.6	
	18*	235.6		58.2	
	19*	231.7		54.6	
	20*	254.7		55.1	
	21*	238.0		50.5	
Post-cut scan	1	135.5	172.5/175.4 ± 21.3	47.3	51.6/53.4 ± 4.7
	2	144.9		47.8	
	3	133.8		50.1	
	4	159.0		60.0	
	5	160.5		49.3	
	6	165.2		53.0	
	7	167.9		57.6	
	8	194.7		65.0	
	10	166.5		48.9	
	11	178.3		49.5	
	12	178.8		61.5	
	16*	177.0		48.3	
	17*	221.8		57.3	
	18	210.0		55.0	
	19	220.0		49.0	
	20*	192.7		55.1	

^*^Consecutive scans were performed under the same conditions. The signal averages of 22 Pre-cut scans of these 5 specimens were taken. The signal averages of 12 Post-cut scans of these 3 specimens were taken (See Table 1)

**Fig. 4:**
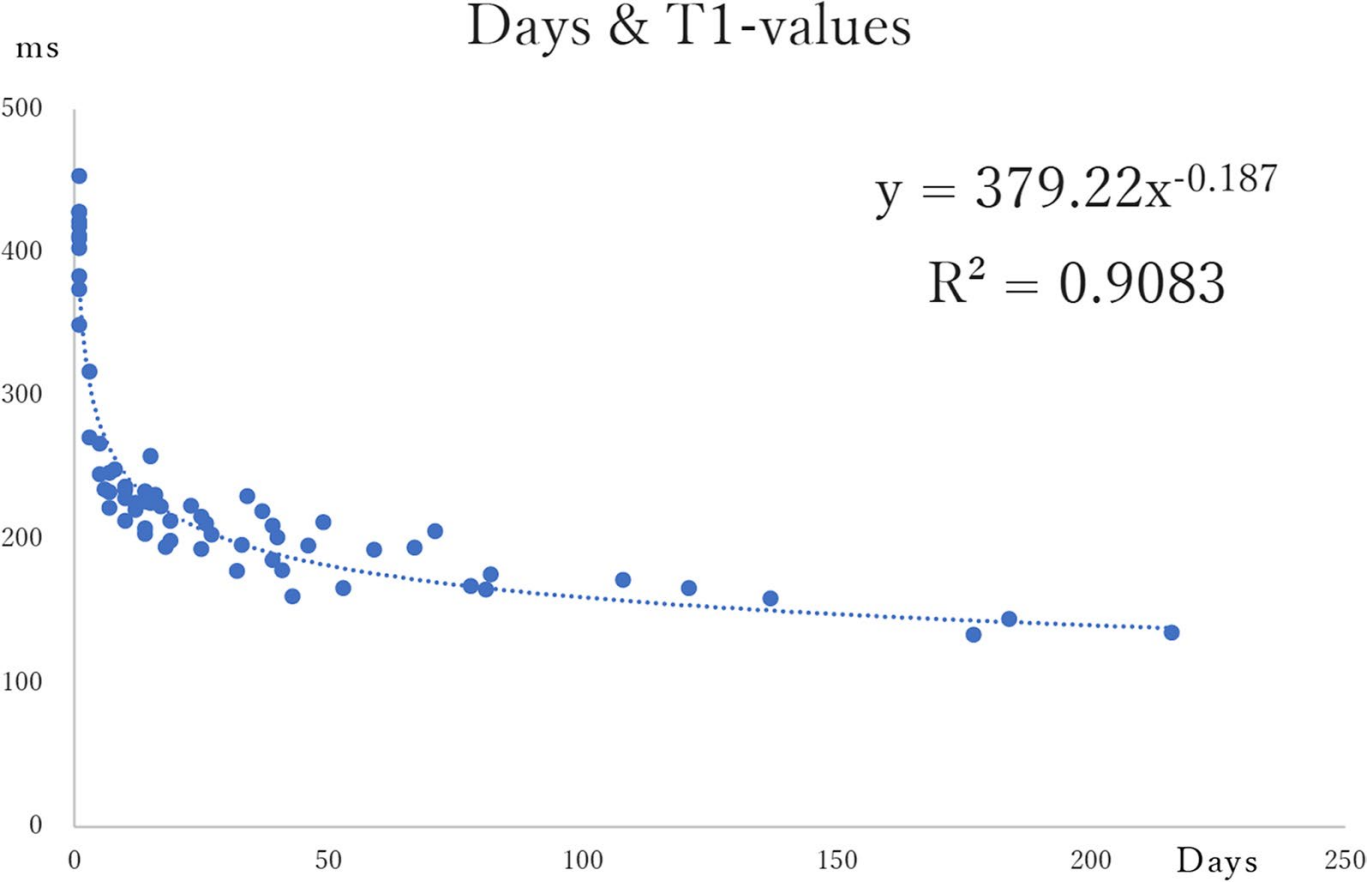
Changes over time in T1-values from organ removal to scanning

**Fig. 5:**
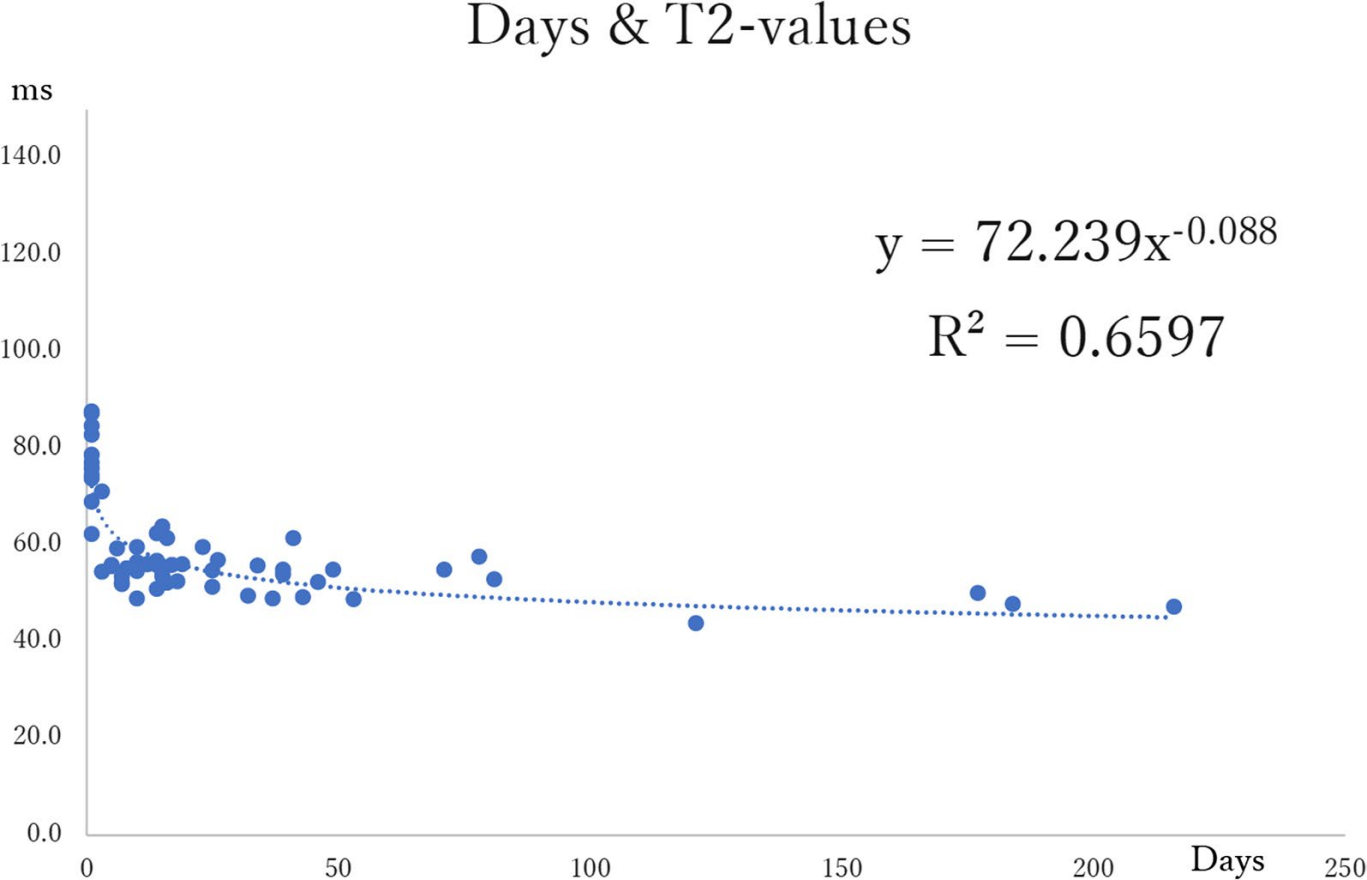
Changes over time in T2-values from organ removal to scanning

The changes over time in T1- and T2-values from organ removal to scanning as sliced specimens were graphed, and the power approximation curves were calculated (Figs. 4, 5). The median T1-values at each measurement time point tended to decrease from immediately after removal, with the median T1-value from cut tissue after formalin fixation shortened to approximately 55% of that immediately after removal. The median T1-value decreased to approximately 42% during the interval from the raw scan immediately after removal to the post-cut scan.

T2-values at each measurement time point tended to decrease from immediately after removal, with the median T2-value from cut tissue after formalin fixation shortened to approximately 70% of that immediately after removal. The median T2-value decreased to approximately 67% during the interval from immediately after removal to MRI scanning of the sliced specimen. The T2-value exhibited the same tendency to decrease as the T1-value, but this tendency was more pronounced for the T1-value.

For the T1-values, the equation derived from all data from the raw scan to the post-cut scan was y = 379.22x^–0.187^, with R^2^ = 0.9083. For the T2-values, the equation derived from all data was y = 72.239x^–0.088^, with R^2^ = 0.6597. There was no specimen in which either the T1 or T2 signal value lengthened over time.

In cases 16 to 21, multiple scans of the same sample were taken. The approximation curves for these multiple scans of the same sample were y = 352.92x−0.16, R^2^ = 0.8522 for T1 and y = 72.045x−0.091, R^2^ = 0.5954 for T2. Although the reliability of both T1- and T2-values was lower than that of the complete data, they did not show significant differences. (Additional file 2: Figures S1, Additional file 3: Fig. S2)

Temperature measurements of each heart before and after each series of scans showed that the temperature of the laboratory did not change by more than 1 °C over this time, and changes in specimen surface temperature were also within the range ± 0.5 °C.

## Discussion

In this study, signal changes in images of specimens were investigated on the basis of data numerically converted from T1- and T2-values. It was found that formalin fixation shortened both T1- and T2-values over time, and approximation formulae were derived to show these decreases over time.

In the present study, it was necessary to cut the specimen at some point and examine the pathological image in order to determine whether it was a normal site. Therefore, it was necessary to cut the heart in order to target the normal part for signal measurement. The signal changes of the specimens after cutting were also measured. In order to observe longer term signal changes, the signal changes of the specimens after cutting were also measured based on the findings from the histopathological specimens.

The heart specimens showed a gradual curve of signal change over time, with no steep signal change due to the continued fixation in formalin solution after cutting.

As yet, no scientific findings that might explain the effect of formalin fixation in shortening T1- and T2-values have been identified. However, formalin fixation inhibits the progression of autolysis and decay that starts the moment tissues are harvested, and the principle of fixation lies in the structure of formaldehyde. When aldehyde groups react with the amine groups on protein side-chains, hydroxymethyl groups, which further react with amine groups to form methylene bridges that stabilize the proteins (bridge fixation), are generated. This bridge formation actually “fixes” proteins so that they are unable to denature. This is believed to make them more stable [42].

The rate at which formalin solution penetrates the tissue has been reported to be 2.4 mm in 24 h for formaldehyde by observation and prediction [43]. The mean thickness of the left ventricular myocardial wall at the ROI site in the present study was 12.66 mm in all patients, and the mean thickness of the myocardial wall at the ROI site was 13.3 mm in men and 11.5 mm in women. Formalin reaches the heart deeply from both the luminal and epicardial sides. In the present study, the shortest period after formalin fixation when MRI was performed was in case 18 and case 20; myocardial wall thickness at the ROI site was 12.0 mm and 13.0 mm, respectively. In both cases, the thickness of the myocardial wall was within 14.4 mm (2.4 mm × 2 × 3 days).

The shortening of T1- and T2-values can be understood as commensurate with a reduction in the water content (water molecules) of the organ concerned. The following are two possible reasons for this reduction in water content.

The first is that tissue generally contracts as a result of fixation with formaldehyde, as described above. Formalin fixation reduces the distance between proteins, so that, although there are differences due to the structural content of particular tissues, in general the tissue contracts, physically expelling the water contained within it, reducing the water content per unit volume of the specimen.

Another possibility is tissue dehydration. The undiluted formaldehyde solution is dissolved in anhydrous to a 35–37% solution. This means that 10% formalin solution contains 10% formaldehyde solution, so a simple formalin fixation solution contains 10% methanol. Because methanol has a powerful dehydrating effect, this may therefore exert a dehydrating action by draining water from the tissue. It is thus a reasonable conjecture that these two effects may act synergistically to shorten the T1- and T2-values.

It was possible to express this shortening of the T1- and T2-values due to formalin fixation in the form of approximation equations, suggesting that, if the time elapsed since the start of formalin fixation is known, it may be possible to estimate the T1- and T2-values of heart tissue without apparent pathological change at the start of formalin fixation. Once the T1- and T2-values for different conditions (diseases), rather than normal tissue, have been measured, MRI signal values may become useful for analyzing pathological conditions at all time points.

In previous studies, the tissue relaxation time depended on the magnetic field, temperature, time after resection, and in vivo or in vitro conditions [44–47]. Since the rates of change of T1 and T2 relaxation times vary depending on the tissue, it has already been pointed out that the contrast of the image varies depending on the organ under study, the condition of the tissue, and the strength of the magnetic field.

The graphs of the T2-values exhibited greater variation than did those for the T1-values. This may have been because the MRI scanner used for measurements had a magnetostatic field strength of 0.3-T, and in light of the fact that the range of the signal measurements that exhibited this variation was within a 10-ms range, a major reason may have been that signal acquisition was being performed in a measurement range close to the limit of accuracy of the MRI scanner for measuring signal values. It is possible that, because the inversion recovery (IR) method was used to measure T1-values from gradually increasing signals, it was possible to obtain higher values, but because the spin echo (SE) method was used to measure T2-values, the fact that faint MRI signals that were gradually decreasing were measured may have caused greater variation of T2-values than of T1-values. One method of ascertaining whether this is the case would be to extend the recovery time (TR) in the SE method and increase the number of measurement points with a long TE, thus decreasing measurement error, but in the present study, MRI signals were measured consecutively over a period of more than 6 h, making it unrealistic to further prolong the TR or increase the number of measurement points. When the variation in T2 signal values is plotted on the vertical scale on the graph, the extent of variation is not very great compared with the T1-values.

Let us consider the graph on which the T2-values are plotted. It is known that the SE method, which is optimized to some extent, has less error in measurement. However, the static magnetic field strength of the MRI system used in this study was 0.3 T. The values obtained at this static field strength of course contain errors due to the multiplication of the noise factor inherent to the machine and the noise factor contained in the sample. The measurement range of the obtained T1-values was about 150 ms to 200 ms in the time phase after the cut. In comparison, the measurement range of the T2-value was very short, about 50 to 60 ms in the time phase after the cut. When the vertical axis scales of the graphs obtained for T1- and T2-values were aligned, the T2-value curve was measured in such a time range that it appeared to be almost linear.

Since this was a 0.3-T MRI system, we believe that it had reached the range where the measurement limit must be considered. The TR for the SE method was set at 3000 ms this time. If the TR is extended further, the resulting signal will be even weaker. The TR time for MRI signal acquisition is determined by considering the reliability of the obtained values. The limit of signal acquisition with a properly chosen SE method also needed to be considered depending on the device.

Temperature changes also reportedly affect MRI signals, [48] but temperature measurements of each heart before and after each series of scans showed that the temperature of the laboratory did not change by more than 1 °C over this time, and changes in specimen surface temperature were also within the range of around 0.5 °C. This indicated that the effect of temperature changes could be excluded.

There were four limitations to this study. The first was that only areas assessed to be normal heart tissue structures on histopathological specimens were investigated. We intend to carry out further studies of diseased areas in the future. The second was that a 0.3-T device was used for the study. However, we consider that data from 0.3-T devices will be important for the practice of PM-MRI on removed hearts in the future. The third was that, although the specimens investigated in this study came from a total of 21 patients, it was possible to carry out MRI scanning at all stages of specimen preparation in only 7, and further studies of larger numbers of cases will be required in the future.

Finally, the temperature changes in the myocardial wall tissue during “raw scan” imaging were not clearly investigated. Temperature measurements were monitored at the beginning and at the end of the scan. Considering the time lapse from the post-extraction state to the start of the scan, it was thought that extreme temperature changes could be avoided.

## Conclusion

MRI signal changes in images of heart specimens were investigated. Formalin fixation shortened both T1- and T2-values over time, and approximation formulae were derived to show these decreases over time.

## Supplementary Information

**Additional file 1**. The cause of death and data on subjects’ cardiovascular risk.

**Additional file 2**. The changes of T1-value in relaxation time of the same specimens from Case16 to 21. The approximate curve of the values obtained by multiple scans with the same sample is shown.

**Additional file 3**. The changes of T2-value in relaxation time of the same specimens from Case16 to 21. The approximate curve of the values obtained by multiple scans with the same sample is shown.

## Data Availability

These data will not be shared due to data protection requirements. The full datasets are not openly available. Data can be obtained on reasonable request from the corresponding author. Information about the data and conditions for access are available from the corresponding author (Kiyokadzu Ebata, ebatakiyokadzu@gmail.com).

## Abbreviations

PM: Postmortem
MRI: Magnetic resonance imaging
PMI: Postmortem imaging
CT: Computed tomography
Ai: Autopsy imaging
US: Ultrasound
T2WI: T2-weighted imaging
IR: Inversion recovery
TI: Inversion time
ms: Milliseconds
TE: Echo time
ROIs: Regions of interest
T1WI: T1-weighted imaging
T2*: T2 star
FLAIR: Fluid-attenuated inversion-recovery
SE: Spin echo
TR: Recovery time
