# Supplementary figures and images for: Changes in magnetic resonance imaging relaxation time on postmortem magnetic resonance imaging of formalin-fixed human normal heart tissue
Source: BMC Med Imaging. 2021 Sep 23;21:134. doi: 10.1186/s12880-021-00666-5 (PMC8459544; doi:10.1186/s12880-021-00666-5)

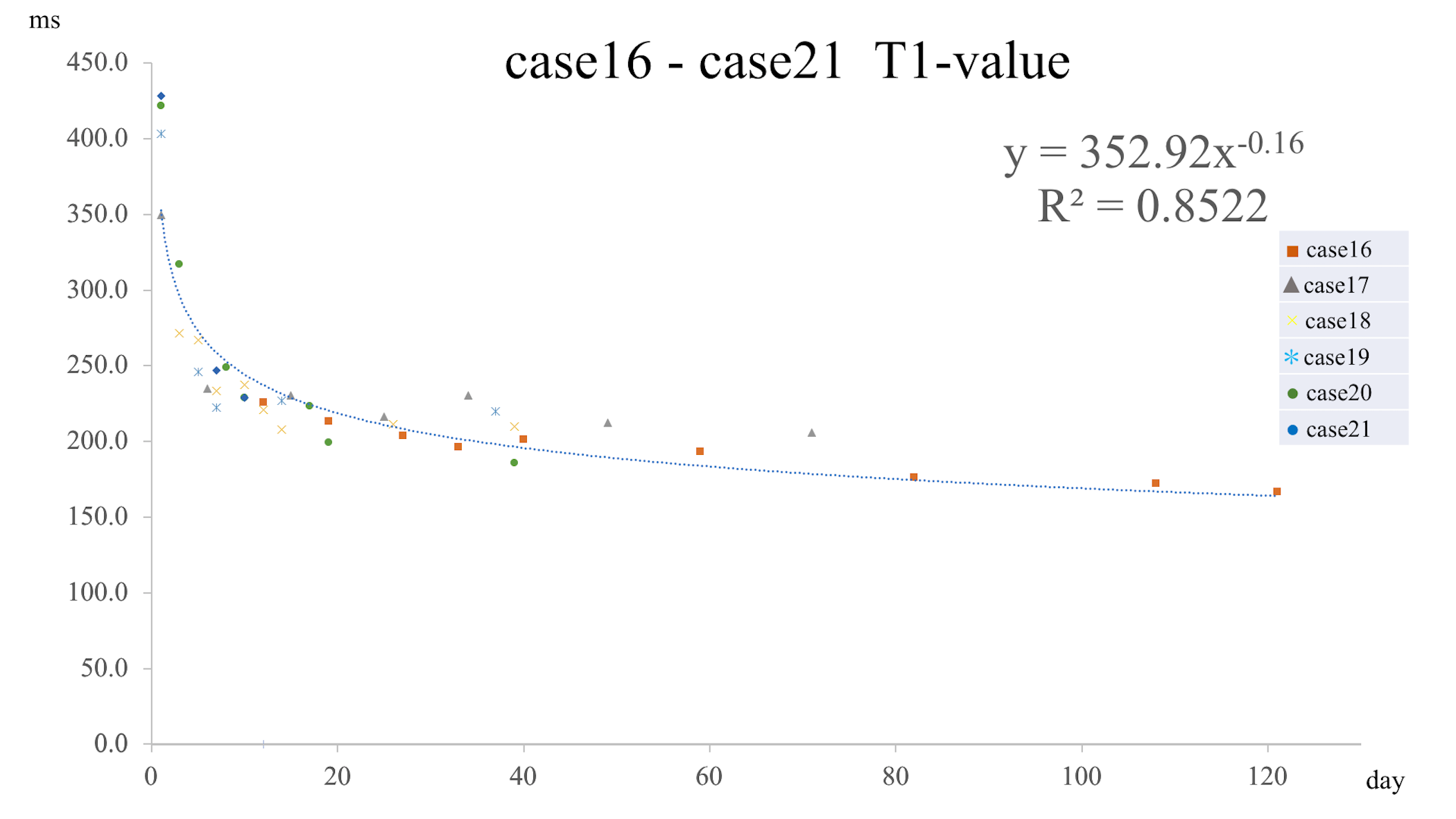

Supplement: Supplementary file 2 — Additional file 2. The changes of T1-value in relaxation time of the same specimens from Case16 to 21. The approximate curve of the values obtained by multiple scans with the same sample is shown. [file 12880_2021_666_MOESM2_ESM.tiff]

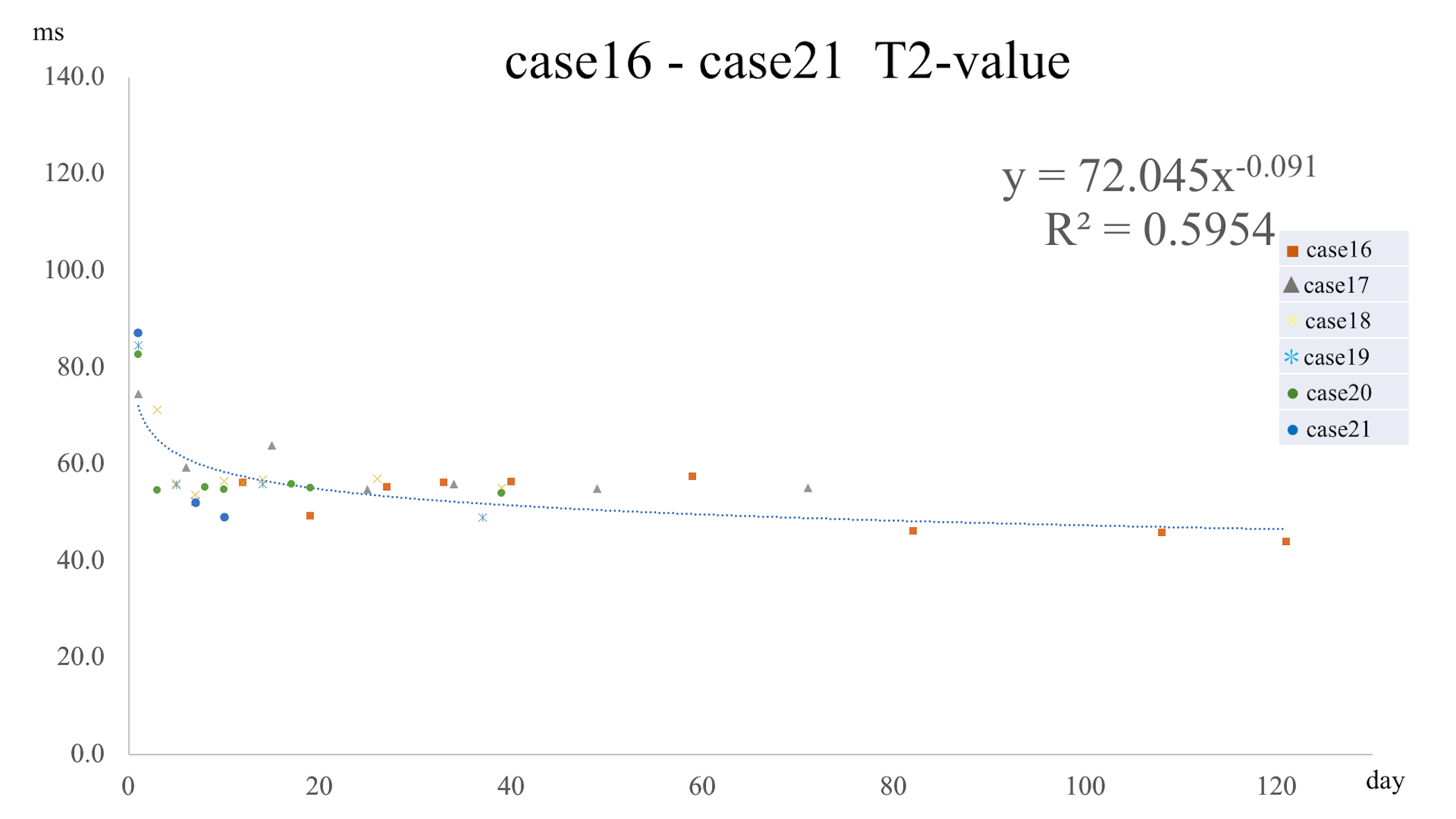

Supplement: Supplementary file 3 — Additional file 3. The changes of T2-value in relaxation time of the same specimens from Case16 to 21. The approximate curve of the values obtained by multiple scans with the same sample is shown. [file 12880_2021_666_MOESM3_ESM.tiff]
